# Supplementary material for: Female-specific myoinhibitory peptide neurons regulate mating receptivity in Drosophila melanogaster
Source: Nat Commun. 2017 Nov 21;8:1630. doi: 10.1038/s41467-017-01794-9 (PMC5696375; doi:10.1038/s41467-017-01794-9)
Supplement: Supplementary file 1 — Supplementary Information [file 41467_2017_1794_MOESM1_ESM.pdf]

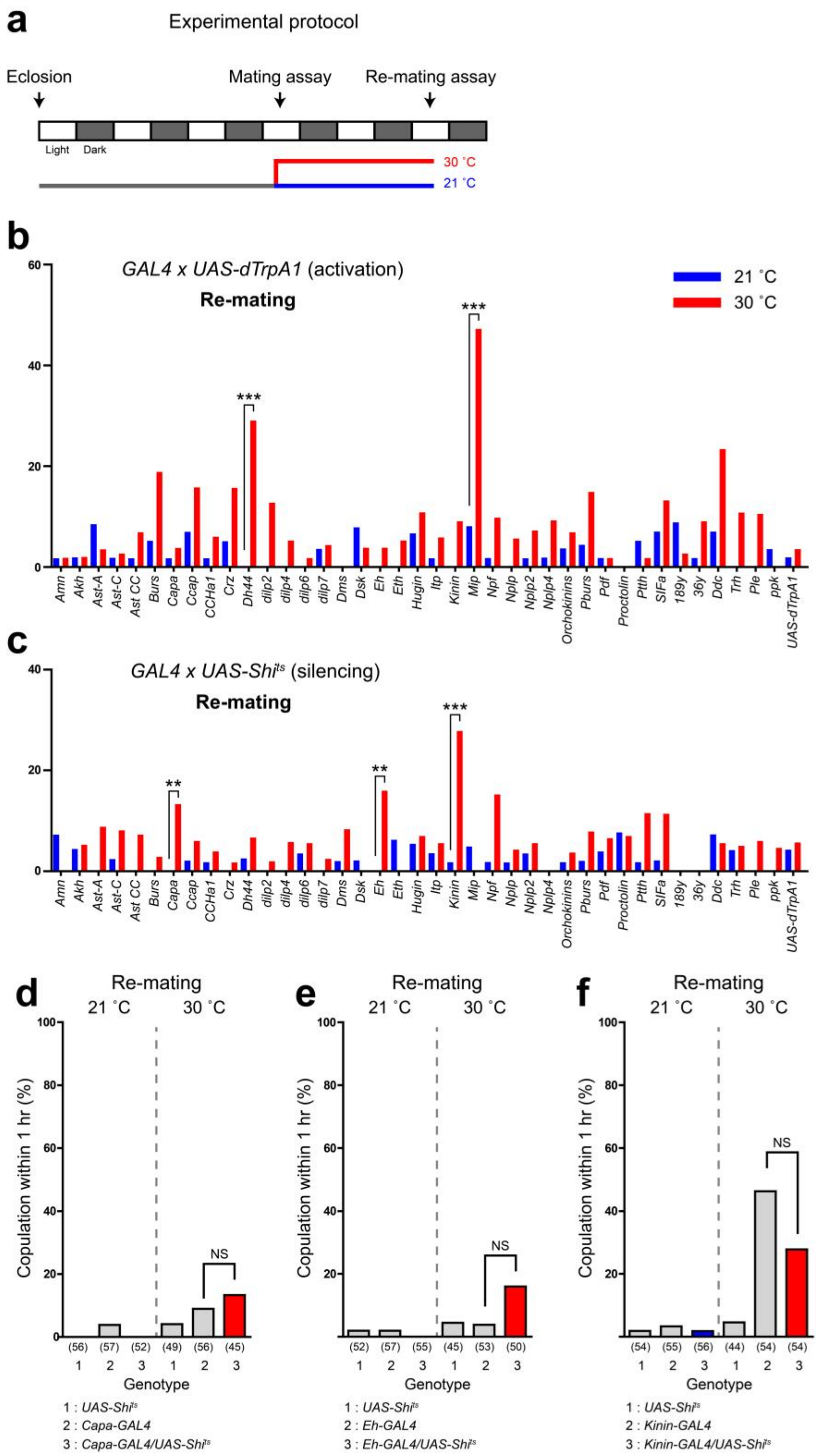

Supplementary Figure 1. A genetic screen for neuromodulator *GAL4* neurons that regulate the re-mating of mated females

(**a**) The experimental procedure used for the behaviour assays. For the re-mating assay, females were mated individually with naïve *CS* males and kept at the indicated temperatures for 48 hr before being paired with a second naïve *CS* male.

(**b-f**) Re-mating frequencies for mated females of the indicated genotypes, scored as the percentage of females that copulate within 1 hr. *n* = 22–59 for each bar. (**b**, **c**). The numbers in parentheses (**d-f**) indicate *n*. NS indicates non-significance ($P > 0.05$); *** $P < 0.001$ for comparisons against the blue bar (**b**, **c**) or the indicated grey bar (**d-f**); Chi-square test.

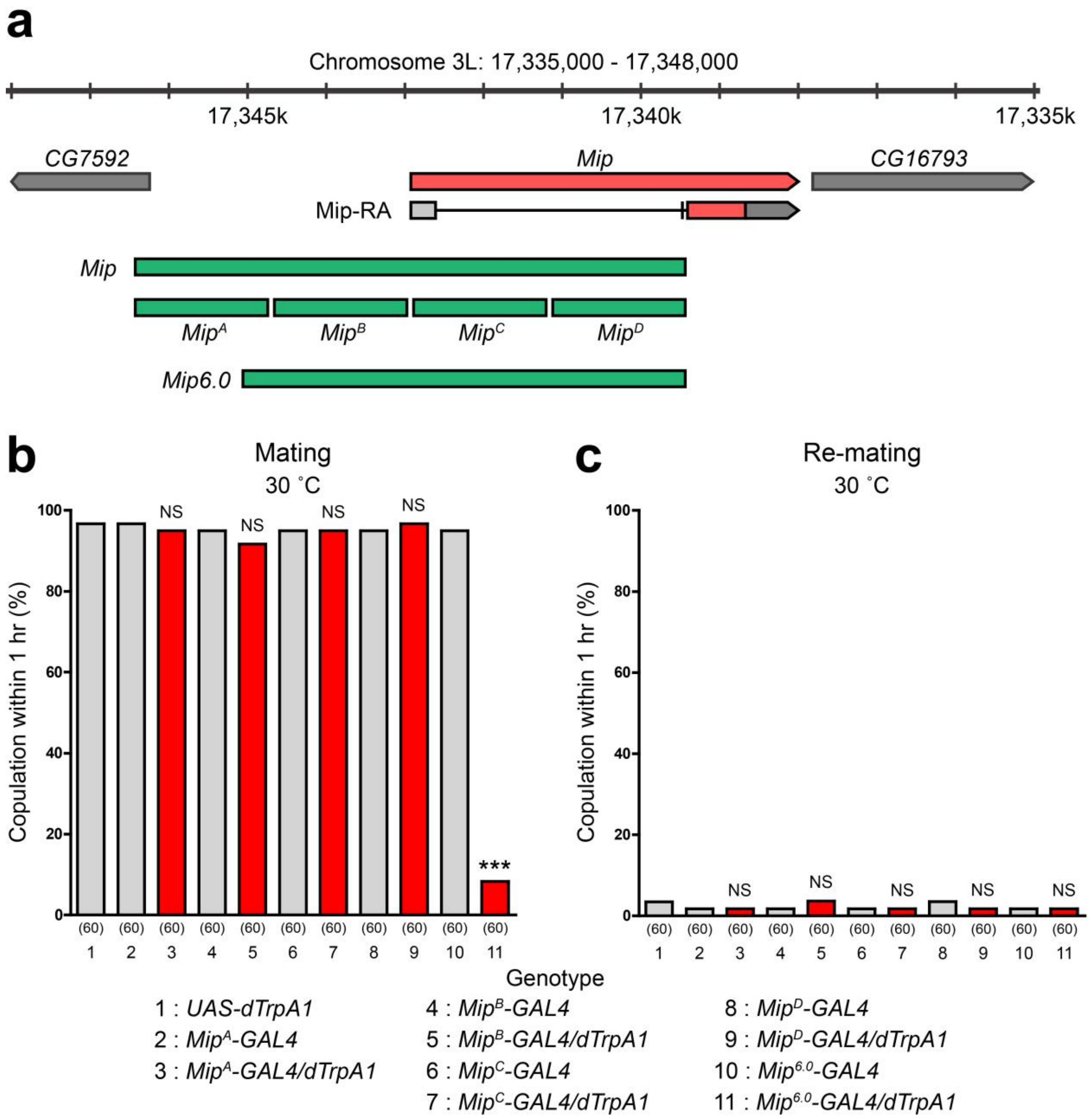

Supplementary Figure 2. Generation of *Mip-GAL4* and *Mip promoter bashing-GAL4* lines and the effects of those activated *GAL4* neurons on mating frequency

(**a**) *Mip* gene structure and the genomic fragments used to generate *Mip-GAL4* and the other related *GAL4* and *GAL80* transgenes (green bars).

(**b**) Mating frequencies of virgin females of the indicated genotypes, scored as the percentage of females that copulate within 1 hr at 30 ^{o}C.

(**c**) Re-mating frequencies of mated females of the indicated genotypes, scored as the percentage of females that copulate within 1 hr at 30 ^{o}C. The numbers in parentheses indicate

n. NS indicates non-significance ($P > 0.05$); *** $P < 0.001$ for comparisons against both controls (grey bars); Chi-square test (**b**, **c**)

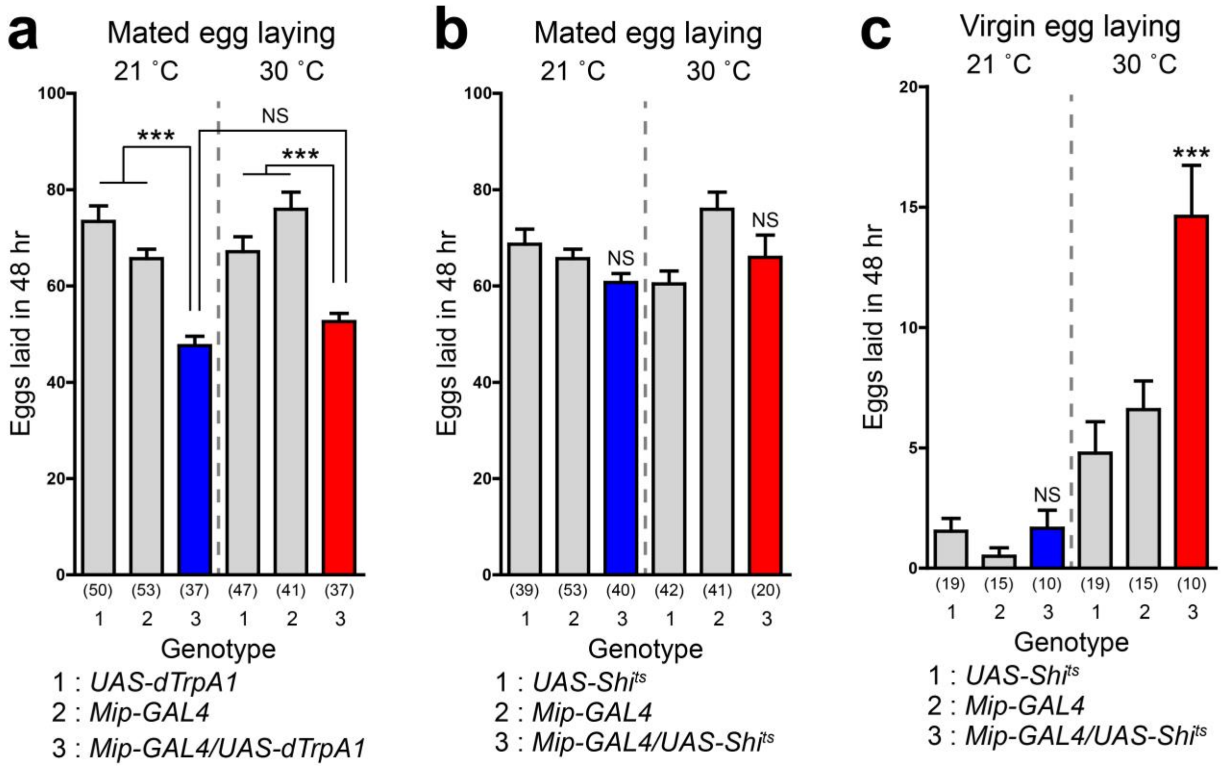

Supplementary Figure 3. Silencing of *Mip* neurons increases virgin egg laying

(**a-c**) The number of eggs laid by mated females (**a**, **b**) or virgin females (**c**) in 48 hr. Data are presented as means ± s.e.m. The numbers in parentheses indicate *n*. NS indicates non-significance ($P > 0.05$); *** $P < 0.001$ for comparisons against the controls (grey bars); one-way ANOVA with Tukey's multiple comparison test.

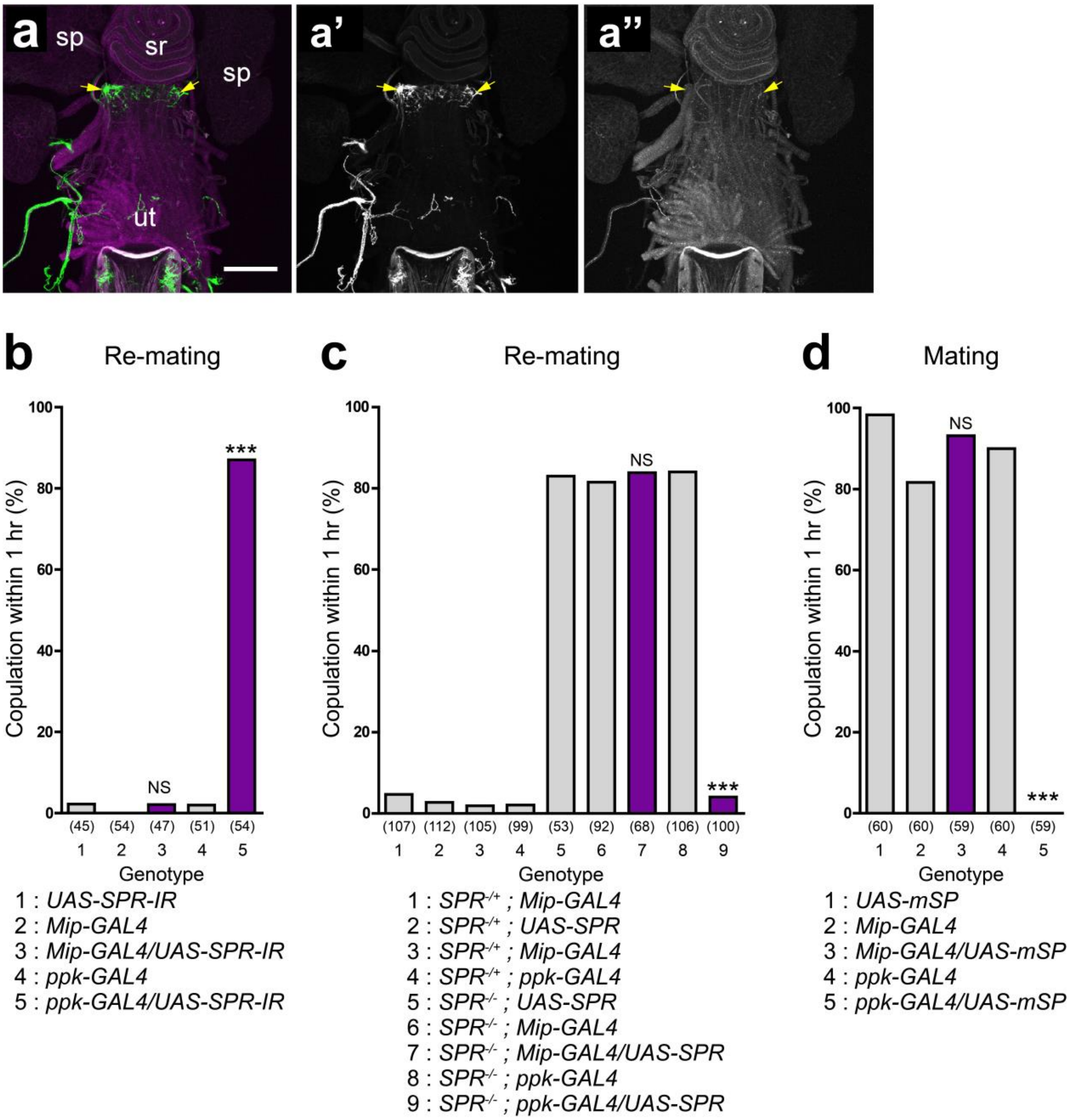

Supplementary Figure 4. *Mip* neurons contain no SPSNs and are not direct targets of SP

(**a**) Confocal sections of the reproductive organs from a *Mip-GAL4, UAS-mCherry, ppk-LexA, LexAop-mCD8-EGFP* female stained with anti-EGFP (green) (**a’**) and anti-RFP (magenta) (**a’’**). Arrows indicate *ppk*-positive SPR neurons (SPSN), which do not express *Mip-GAL4*. sp, spermathecae; sr, seminal receptacle; ut, uterus. Scale bar, 50 µm.

(**b**, **c**) Re-mating frequencies of mated females of the indicated genotypes.

(**d**) Mating frequencies of virgin females of the indicated genotypes. The numbers in parentheses indicate *n*. NS indicates non-significance ($P > 0.05$); *** $P < 0.001$ for comparisons against the controls (grey bars); Chi-square test.

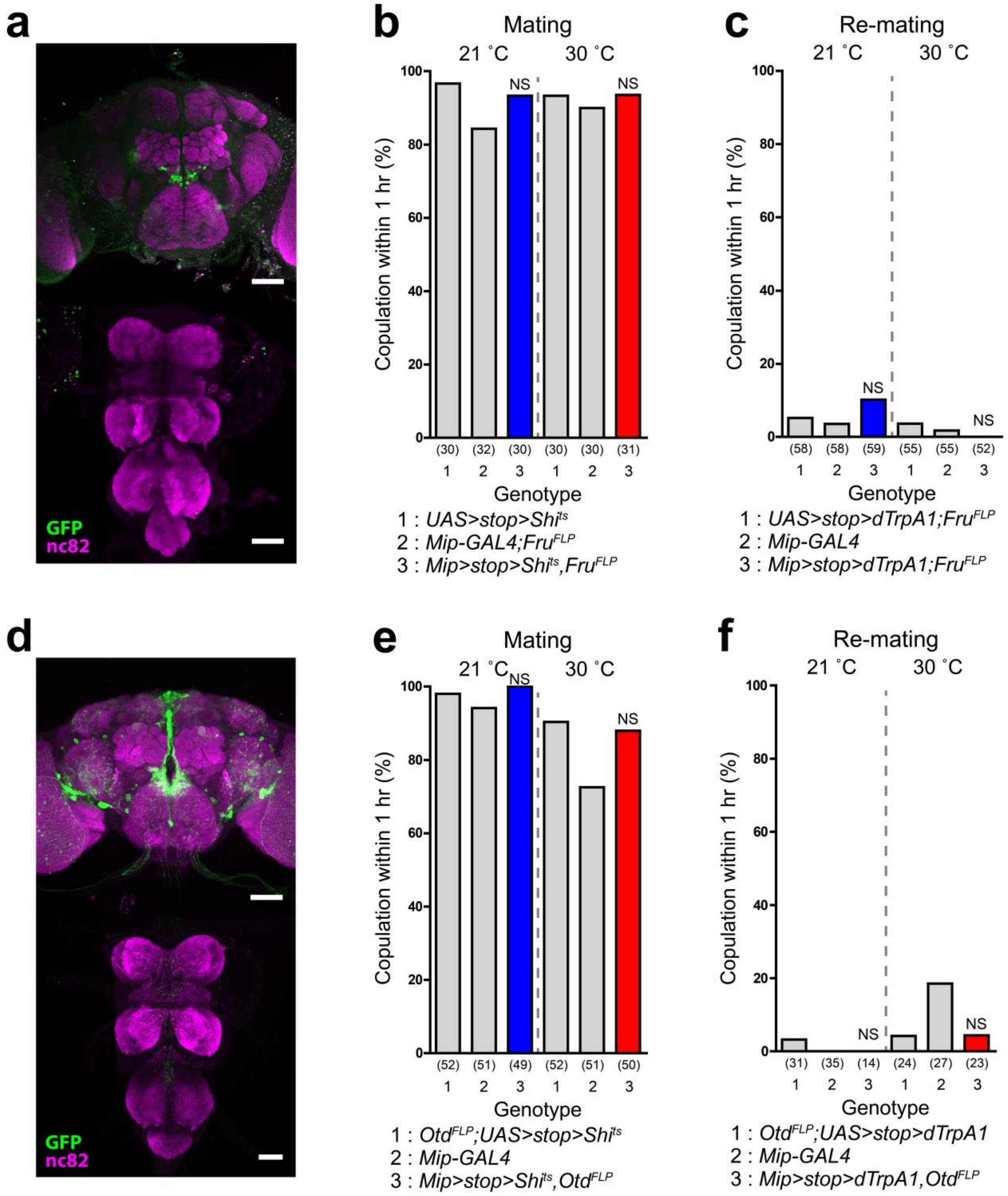

Supplementary Figure 5. Intersectional genetic analyses of *Mip* neurons

(**a**) *Mip* and *fru* double-positive cells in the CNS of a female carrying *Mip-GAL4, UAS>stop>mCD8-GFP* and *fru*^{FLP} stained with anti-GFP (green) and anti-nc82 (magenta) antibodies. Scale bars, 50 µm.

(**b**, **e**) Mating frequencies of virgin females of the indicated genotypes, scored as the percentage of females that copulate within 1 hr.

(**d**) *Mip* and *Otd* double-positive cells in the CNS of a female carrying *Mip-GAL4, UAS>stop>mCD8-GFP* and Otd^{FLP} stained with anti-GFP (green) and anti-nc82 (magenta) antibodies. Note the absence of labelled somas in the VNC. Scale bars, 50 μm.

(**c**, **f**) Re-mating frequencies of mated females. The numbers in parentheses indicate *n*. NS indicates non-significance ($P > 0.05$); *** $P < 0.001$ for comparisons against the controls (grey bars); Chi-square test.

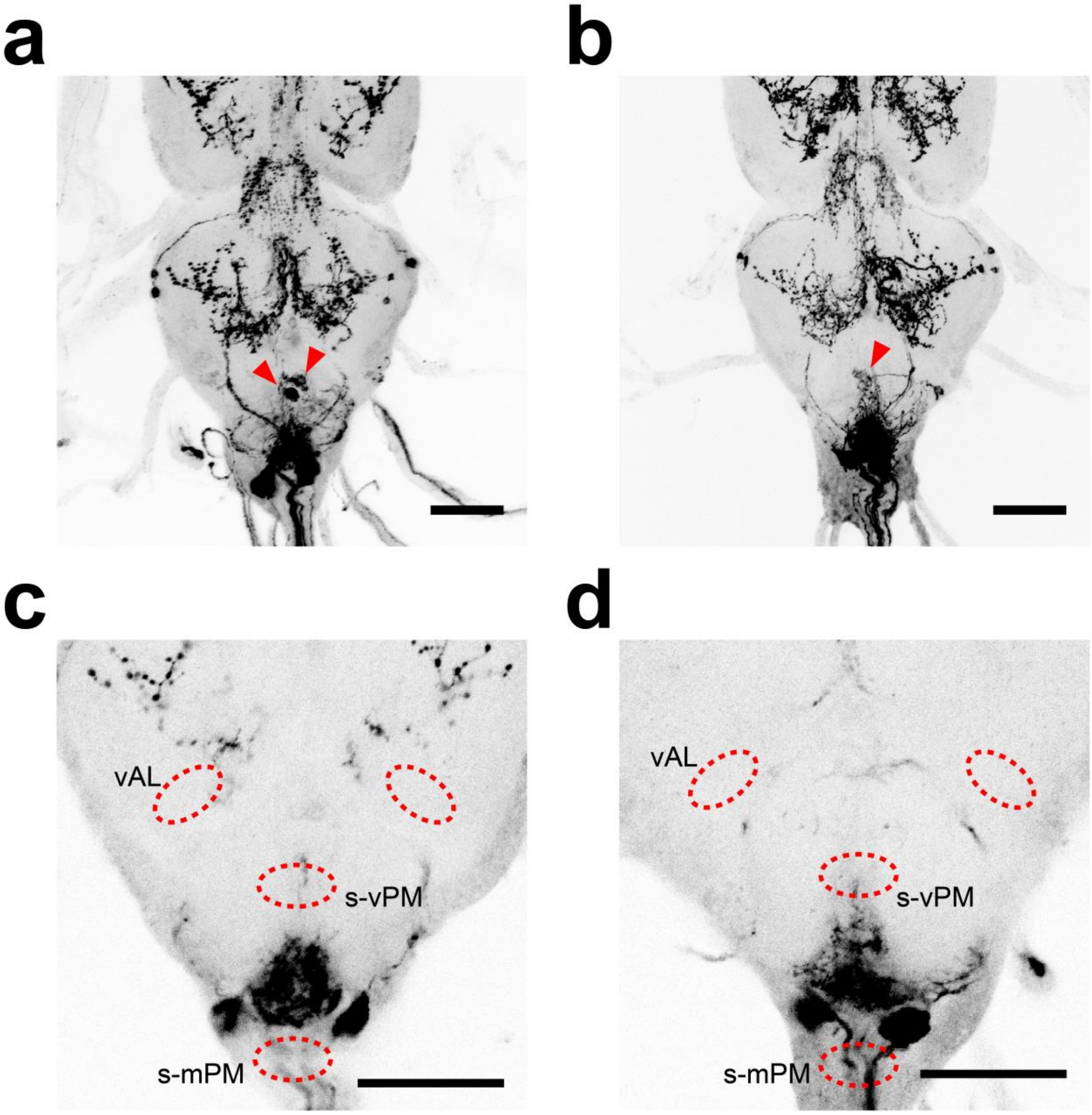

Supplementary Figure 6. Increased Ca^{2+} activity in *vAM* neurons

(**a**-**d**) Negative images of TRIC labelling (anti-GFP) in the AGs of virgin (**a**, **c**) and mated females (**b**, **d**). Arrowheads indicate *vAM* somas. TRIC does not stain cells in locations (dotted circles) where indicated neurons occur in both virgin (**c**) and mated females (**d**). Scale bars, 50 µm.
