# Supplementary material for: Female-specific myoinhibitory peptide neurons regulate mating receptivity in Drosophila melanogaster
Source: Nat Commun. 2017 Nov 21;8:1630. doi: 10.1038/s41467-017-01794-9 (PMC5696375; doi:10.1038/s41467-017-01794-9)
Supplement: Supplementary file 3 — Description of Additional Supplementary Files [file 41467_2017_1794_MOESM3_ESM.pdf]

File Name: Supplementary Movie 1

Description: A video clip showing 3-day-old virgin females of the indicated genotypes paired with 4-day-old naïve *CS* males at two temperature conditions. At 30 ^{o}C, the virgin female carrying *Mip-GAL4, Mip6.0-GAL80* and *UAS-Shi*^{ts} performs frequent ovipositor extrusions in response to male advances. At 21°C, however, she performs no ovipositor extrusions in the presence of an actively courting male, permitting copulation by the end of the video clip.
